# Draft Genome Sequence of *Corynebacterium ulcerans* Strain 04-3911, Isolated from Humans

**DOI:** 10.1128/genomeA.00171-16

**Published:** 2016-03-31

**Authors:** Luis C. Guimarães, Marcus V. C. Viana, Leandro J. Benevides, Diego C. B. Mariano, Adooney A. O. Veras, Pablo H. C. Sá, Flávia S. Rocha, Priscilla C. B. Vilas Boas, Siomar C. Soares, Maria S. Barbosa, Nicole Guiso, Edgar Badell, Adriana R. Carneiro, Vasco Azevedo, Rommel T. J. Ramos, Artur Silva

**Affiliations:** a Institute of Biological Sciences, Federal University of Pará (UFPA), Belém, Pará, Brazil; b Institute of Biological Sciences, Federal University of Minas Gerais (UFMG), Belo Horizonte, Minas Gerais, Brazil; c Department of Immunology, Microbiology and Parasitology, Institute of Biological Sciences and Natural Sciences, Federal University of Triângulo Mineiro (UFTM), Uberaba, Minas Gerais, Brazil; d Institut Pasteur, Unité de Prévention et Thérapies Moléculaires des Maladies Humaines, National Centre of Reference of Toxigenic Corynebacteria, Paris, France

## Abstract

*Corynebacterium ulcerans* is a pathogenic bacterium infecting wild and domesticated animals; some infection cases in humans have increased throughout the world. The current study describes the draft genome of strain 04-3911, isolated from humans. The draft genome has 2,492,680 bp, 2,143 coding sequences, 12 rRNA genes, and 50 tRNA genes.

## GENOME ANNOUNCEMENT

*Corynebacterium ulcerans* belongs to a suprageneric group of actinomycetes that also includes the genera *Mycobacterium*, *Nocardia*, and *Rhodococcus*, termed the CMNR group (*Corynebacterium*, *Mycobacterium*, *Nocardia*, and *Rhodococcus*). This bacterium is facultative anaerobic, nonsporulating, nonmotile, catalase positive, and nitrate and oxidase negative (1, 2). Analyses of 16S rRNA gene sequences showed that *C. ulcerans*, *Corynebacterium pseudotuberculosis*, and *Corynebacterium diphtheriae* are closely related (2). Otherwise, there are *C. ulcerans* strains coding for a diphtheria toxin similar to that encoded by toxigenic strains of *Corynebacterium diphtheriae* (3), as well as *C. ulcerans* strains coding for a dermonecrotic toxin with similarity to toxic phospholipase D (PLD) from *C. pseudotuberculosis* (4).

*C. ulcerans* has been detected in a variety of wild and domesticated animals, suggesting that both groups may attend as reservoirs for zoonotic transmission (5). This bacterium is an emergent pathogen due its frequency and severity of human infections reported during the last two decades in various countries (6). In humans, it causes diphtheria-like disease, pharyngitis, sinusitis, tonsillitis, pulmonary nodules, and skin ulcers (7). Here, we present the draft genome sequence of *C. ulcerans* 04-3911, isolated from humans. The genome sequencing was performed by the SOLiD platform, using a fragment library. The predicted genome coverage was approximately 6,000×, based on *C. ulcerans* genomes available in GenBank (http://www.ncbi.nlm.nih.gov/genbank/). The software Velvet version 1.2.10 (8) was used with a *de novo*-assembled strategy. The assembly generated 40 contigs, with 2,492,680 bp, which were submitted to GenBank to automatic annotation. The genome has 2,143 coding sequences, 12 rRNA genes, 50 tRNA genes, 55 pseudogenes, 2 clustered regularly interspaced short palindromic repeat (CRISPR) arrays, and a G+C content of 53.30%. This genome is part of further studies of comparative genomics, pathogenicity, and vaccine and drug targets of the species.

### Nucleotide sequence accession numbers.

The *C. ulcerans* whole-genome shotgun (WGS) project has the project accession no. LGSX00000000. The version described in this paper is version LGSX01000000 and consists of sequences LGSX01000001 to LGSX01000040.
